# Nonverbal Action Interpretation Guides Novel Word Disambiguation in 12-Month-Olds

**DOI:** 10.1162/opmi_a_00055

**Published:** 2022-07-01

**Authors:** Barbara Pomiechowska, Gergely Csibra

**Affiliations:** Cognitive Development Center, Department of Cognitive Science, Central European University; Department of Psychological Sciences, Birkbeck, University of London

**Keywords:** action interpretation, word learning, reference, cognitive development, mutual exclusivity

## Abstract

Whether young infants can exploit sociopragmatic information to interpret new words is a matter of debate. Based on findings and theories from the action interpretation literature, we hypothesized that 12-month-olds should distinguish communicative object-directed actions expressing reference from instrumental object-directed actions indicative of one’s goals, and selectively use the former to identify referents of novel linguistic expressions. This hypothesis was tested across four eye-tracking experiments. Infants watched pairs of unfamiliar objects, one of which was first targeted by either a communicative action (e.g., pointing) or an instrumental action (e.g., grasping) and then labeled with a novel word. As predicted, infants fast-mapped the novel words onto the targeted objects after pointing (Experiments 1 and 4) but not after grasping (Experiment 2) unless the grasping action was preceded by an ostensive signal (Experiment 3). Moreover, whenever infants mapped a novel word onto the object indicated by a communicative action, they tended to map a different novel word onto the distractor object, displaying a *mutual exclusivity* effect. This reliance on nonverbal action interpretation in the disambiguation of novel words indicates that sociopragmatic inferences about reference likely supplement associative and statistical learning mechanisms from the outset of word learning.

## INTRODUCTION

Infants begin to learn words during the first year of life (Bergelson & Aslin, 2017a, 2017b; Bergelson & Swingley, 2012; Parise & Csibra, 2012). How do they come to ground linguistic symbols in nonlinguistic reality? One of the first steps in this process is to identify arbitrary relationships between linguistic expressions and their immediate referents. While it is generally agreed that referent selection in older children relies heavily on sociopragmatic information (for reviews, Baldwin & Moses, 2001; Golinkoff & Hirsh-Pasek, 2006; Hirsh-Pasek et al., 2004), it remains a contentious issue whether such information is also used by young infants who might lack sophisticated understanding of others’ communicative intentions and practices.

Some argue that, at the beginning of word learning, the ability to set up links between words and objects is the product of domain-general associative and statistical learning mechanisms that track regularities present in one’s environment (Plunkett, 1997; Romberg & Saffran, 2010; Smith, 1999, 2000). Indeed, by 12 months of age, infants can link word forms to salient visual percepts that co-occur with them (Smith & Yu, 2008; Taxitari et al., 2020), and the frequency of co-occurrence determines the strength of the encoded association (Smith et al., 2014; cf. Gleitman & Trueswell, 2020). Others suggest that, like older children, infants should be able to consider the social context in which language is produced to decipher what aspects of the environment might be referred to by the speakers (Akhtar & Tomasello, 2000; Baldwin & Moses, 2001; Tomasello, 2008). Evidence from the literature on action interpretation indicates that, by their first birthday, infants have access to concepts and rich inferential mechanisms that could guide their discovery of word–object relationships expressed in language. However, experimental evidence exploring this hypothesis is scarce and not conclusive.

Not all actions performed by others warrant referent search. Some are carried out toward purely instrumental goals of changing the state of the world (e.g., lifting a cup to pour coffee into it), some for epistemic purposes (e.g., lifting a cup to check whether it is filled), and yet others for communicative purposes of transmitting information to others (e.g., lifting a cup to signal to the waiter that one would like a refill). While these three types of actions can (and often do) involve external objects, these objects serve different roles. In particular, only in communication they function as the immediate referents of the accompanying actions. Thus, while trying to make sense of others’ behavior, people should look for referents only for actions they regard as communicative.

How to tell what goal motivates an observed action? Although action interpretation often involves reasoning about the actor’s mental states (e.g., intentions, desires, beliefs) that might not be available in infancy, it can also be carried out in a nonmentalistic way (Gergely & Csibra, 2003; Jara-Ettinger et al., 2020). Mechanisms to assess what general purpose an action serves include evaluating its means-end structure (Gergely & Csibra, 2003) and matching it against known action concepts (or schemas, e.g., giving: Tatone et al., 2015, 2019; Yin et al., 2020; chasing: Frankenhuis & Barrett, 2013). Additionally, a particular action interpretation can be automatically activated by a trigger stimulus (Csibra, 2003, 2010). For example, ostensive signals naturally render others’ behavior communicative (Sperber & Wilson, 1986), such that instrumental actions become interpreted as pedagogical demonstrations (Hernik & Csibra, 2015; see also Pomiechowska & Csibra, 2017).

Developmental evidence indicates that infants benefit from nonmentalistic action interpretation systems that enable them to interpret certain object-directed actions as instrumental toward specific goals (e.g., approach: Csibra et al., 1999; Gergely et al., 1995; Skerry et al., 2013; Woodward, 1998; Woodward & Guajardo, 2002) and also distinguish them from communicative object-directed actions (e.g., deictic reference: Hernik & Broesch, 2019; Senju & Csibra, 2008; for reviews: Csibra, 2003; Gergely & Jacob, 2012). Therefore, early on, infants have access to inferential tools necessary to solve two major tasks: (1) recognize that only some nonverbal actions require referent identification, and (2) conceive objects targeted by these actions as potential immediate referents.

Studies demonstrate that by 12 months of age communicative gaze (Csibra & Volein, 2008; Ishikawa & Itakura, 2019; Okumura et al., 2013a; Senju & Csibra, 2008) and pointing (Behne et al., 2012; Daum et al., 2013; Pätzold & Liszkowski, 2019) are expected to be directed at objects and enhance their encoding (gaze: Okumura et al., 2013b, 2020; Reid & Striano, 2005; pointing: Hirai & Kanakogi, 2019; Yoon et al., 2008). It remains unclear, however, whether the target objects are conceptualized as referents. Supporting this interpretation, Gliga and Csibra (2009) found that one-year-olds expect familiar kind labels (e.g., “cup,” “duck”) to co-refer with simultaneously produced head turns, gaze shifts, and pointing. That is, they assume that the label should pick out the object indicated by the direction of gaze and pointing. However, no studies to date have provided evidence that the expectation of co-reference between words and actions contributes to referent selection for *novel words* (Hollich et al., 2000). Although 12- to 13-month-olds were shown to acquire word-object mappings coupled with communicative actions (Tsuji et al., 2020; Woodward et al., 1994; cf. Yurovsky & Frank, 2017), their performance could be explained without appealing to action interpretation or reference. Since even noncommunicative object-directed actions orient infants’ attention toward targeted items (Daum et al., 2009; Daum & Gredebäck, 2011), successful word mapping following gaze shifts or pointing might have been supported solely by the formation of associative links between stimuli that co-occur (i.e., the attended objects and the concurrently uttered labels).

To assess the role of action interpretation in referent selection, we investigated whether the nature of an object-directed action that accompanied labeling would influence how infants map novel words to objects. In our study, labeling was accompanied either by pointing or grasping. These actions share essential structural and basic kinematic properties while normally serving distinct functions. Structurally, they can be described as transitive (or object-directed). Kinematically, both involve an arm protrusion away from the body. Functionally, however, pointing is a prototypical communicative action aimed at highlighting some aspects of the environment that are relevant to the speaker’s informative intention (Kita, 2003; Tomasello, 2008), while grasping is a prototypical instrumental action aimed at establishing physical contact with objects in order to retrieve or manipulate them (Woodward, 1998). Although grasping can be performed to express communicative intentions (e.g., when one grasps an object to demonstrate its function or properties), it is most often performed purely instrumentally to realize a goal. Conversely, pointing is rarely performed outside communication, and is universally perceived as a communicative act, whether accompanied by speech or replacing it (Kita, 2003; Tomasello, 2008). In adults, this functional divide between pointing and grasping is reflected in distinct patterns of neural responses triggered by observation of these actions (Pomiechowska & Csibra, 2017).

Across four eye-tracking experiments, infants could map novel labels to unfamiliar objects in one of two ways: by relying either on the interpretation of preceding actions or by picking up on the spatiotemporal contiguity between the moving hand and the object it approached. The action that preceded labeling was either communicative, designating the highlighted object as the referent (e.g., pointing) or instrumental, flagging the target as relevant for the goal of the action (e.g., grasping). If infants rely on action interpretation, they should search for a referent only when labeling was accompanied by a communicative action, such as pointing. Upon finding a referent, the expectation of co-reference between pointing and labeling should lead them to map the novel label onto the object identified as the referent (Gliga & Csibra, 2009). In contrast, observing a noncommunicative instrumental action, such as grasping, should leave infants without a reason to map the label onto the target object. Alternatively, if infants rely on spatiotemporal contiguity between events without engaging in action interpretation, they should map labels onto targeted objects regardless of which action they observed because both highlight a specific object in the scene.

We tested 12-month-olds because they appreciate the structural properties of both pointing and grasping, as evidenced by their expectation that these actions are transitive and object-directed, whether the target object is visible or occluded (pointing: Behne et al., 2012; Daum et al., 2013; Gliga & Csibra, 2009; Pätzold & Liszkowski, 2019; grasping: Daum et al., 2009; Southgate et al., 2010). By this age, infants are also proficient at executing these actions (grasping: von Hofsten & Rönnqvist, 1988; pointing: Carpenter et al., 1998; Leung & Rheingold, 1981), therefore having (literally) firsthand experience with planning, performing, and monitoring of both actions. Concerning functional differences, infants selectively use pointing to direct others’ attention toward objects and events about which they would like to provide (Liszkowski et al., 2006) or receive (Kovács et al., 2014; see also Begus & Southgate, 2012; Liszkowski et al., 2004) information. Therefore, in production, they appreciate the communicative significance of pointing. Finally, infants at this age rapidly form associations between words and objects trained in lab settings when working memory demands are minimized (Pomiechowska & Gliga, 2019; Tsuji et al., 2020).

## EXPERIMENT 1

Experiment 1 investigated whether 12-month-olds rely on pointing to identify the referents of new words. On each trial, infants were first presented with two novel objects. Then, they saw a hand pointing to one of them before hearing an unfamiliar word (e.g., *A moxi!*). These events were immediately followed by a looking-while-listening test (Fernald et al., 2008; Golinkoff et al., 2013), during which infants heard either the trained label (e.g., *Where is the moxi?*) or a novel one (e.g., *Where is the blicket?*). We expected that identifying the object targeted by pointing as the referent of both the action and the co-occurring word would result in a word-object mapping. If infants interpret pointing as a communicative action affording referent assignment and co-reference with concurrent speech, they should look longer at the target object selectively upon hearing the trained, but not the novel, label.

## METHODS

Our stimuli, data, and analysis code are available on OSF (https://osf.io/f4q62/). All analyses were conducted in R (R Core Team, 2020). Mixed-effects models were fitted using lme4 package (Bates et al., 2012).

### Participants

Sixteen healthy full-term 12-month-olds participated in this experiment (mean: 12 months 4 days, range: 11 months 16 days to 12 months 28 days, see section SM1. Sample size in the Supplemental Materials for details on how the current sample size was determined). Seven more infants were tested but excluded from the analysis because they failed to complete the task (*n* = 3), did not provide enough data (*n* = 3), or were short-sighted (*n* = 1). The current task was piloted on an additional 10 infants. No piloting was involved in the subsequent experiments. All infants came from monolingual Hungarian-speaking families. All parents gave informed consent. Infants received a small gift for their participation.

### Apparatus

Binocular gaze data were collected with a TOBII X60 eye tracker (sampling rate: 60 Hz). The visual stimuli were displayed on a 23-inch monitor (sampling rate: 60 Hz, resolution: 1920 × 1080 px). The audio stimuli were delivered via loudspeakers placed directly behind the monitor. The stimuli presentation and data collection were administered using Matlab 2014b, Psychophysics Toolbox PTB-3 (Brainard, 1997; https://psychtoolbox.org/), and Tobii Pro Analytics software development kit (https://www.tobiipro.com/product-listing/tobii-pro-analytics-sdk/).

### Stimuli

#### Objects.

We used eight pairs of novel objects designed specifically for the current study. Items within each pair differed in shape and color to facilitate perceptual discrimination. The pairs of objects were used to create 8 experimental videos (see Design and Procedure).

#### Speech.

We used eight pairs of CVCV (consonant vowel) pseudo-words consistent with Hungarian phonotactics: bite - gupa, /b**i**t**ɛ**/ - /g**u**p**ɒ**/, tegi - kabó, **/tɛgi/ - /kɒboː/**, düpi - baku, **/dypi/ - /bɒku/**, pádu - géte, **/paːdu/ - /geːtɛ/**, kitő - püke, **/kitøː/ - /pykɛ/**, toda - bóta, **/todɒ/ - /boːtɒ/**, fego - mize, **/fɛgo/ - /mizɛ/**, nala - baku, **/nɒlɒ/ - /bɒku/**.

The labels were embedded in carrier phrases: “*Egy [label]. Hú, egy [label]! Egy [label].*” (A [label]. Wow, a [label]! A [label].), during the naming event, and “*Nézd csak, hol van a [label]? [Label]. [Label].*” (Look, where is the [Label]? [Label]. [Label].) during the test phase. The stimuli were recorded by a female native speaker of Hungarian using infant-directed prosody.

### Design

**Figure 1.**
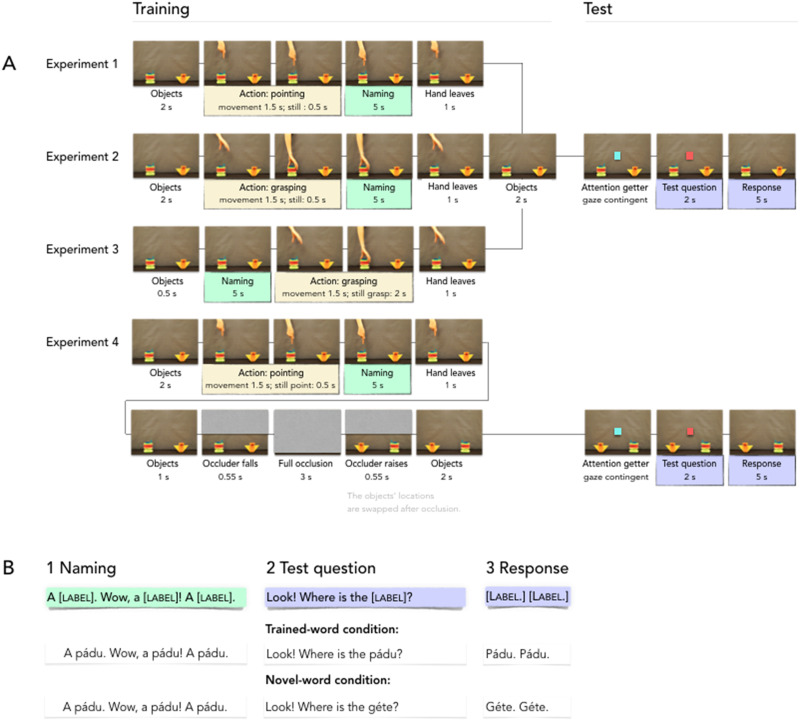
**Schematic of the experimental design.** **A)** Trial structure across Experiments 1–4. While all trials within each experiment had the same structure, each featured a different pair of objects and different pseudo-words. **B)** Examples of speech stimuli at training (B1) and test (B2: test question; B3: response phase). At test, we manipulated whether infants heard the same word as at training (trained-word condition) or a novel previously unheard word (novel-word condition).

The experiment consisted of eight trials. Each trial had the same structure: a *training* immediately followed by a *test* (Figure 1) but involved a different pair of novel objects and word forms. Objects were located on the opposite sides of an otherwise empty table set against a grey background. During *training*, the objects were displayed for 2 seconds before the action started (i.e., before the first frame during which the hand became visible). Then, a downward-pointing hand appeared above one of the objects (target), moved down (2 s), and remained still while the naming stimuli (e.g., “A mize. Wow, a mize! A mize.”) were delivered (5 s; the speech started 0.5 s after the pointing action was completed). After the offset of the speech, the hand moved up and out of the display (1 s); the objects remained on the table (2 s, pretest period).

The test phase followed without interruption: a centrally located dynamic gaze-contingent attention getter appeared between the objects to direct infants’ attention to a neutral point equidistant from both objects (after Yin & Csibra, 2015). The attention getter was accompanied by a jingle (0.4 s). Fixating the attention getter for 0.5 seconds started the looking-while-listening test: the attention getter changed its color, and the test question was played (e.g., “*Look! Where is the mize?*” 2 s).^1^ The disappearance of the attention getter, timed to the offset of the test question, started the test response period (5 s), during which the label was repeated two more times (e.g., “*Mize. Mize.*,” onsets at 1.5 s and 3.5 s). On half of the trials, the test question contained the word introduced in the training phase (e.g., “*mize*”; trained-word condition) and the other half employed a novel, previously unheard word (e.g., “*fegu*”; novel-word condition).

The order of test questions (trained v. novel word), the location of the objects (left v. right), and the action side (left v. right) were randomized by shuffling. Additionally, we counterbalanced across participants the assignment between object pairs and word pairs, and the order in which they were presented.

### Procedure

Testing took place in a soundproof room. Infants were seated on their parent’s lap, approximately 60 cm away from the monitor. The parents wore opaque glasses and were instructed to remain silent and withdraw from interacting with the infants. A 5-point infant-friendly calibration preceded the experimental task and was repeated until at least 4 points were successfully calibrated.

### Data Analysis

To be included in the final analysis infants had to contribute a minimum of two valid trials per condition. We considered valid a trial with at least 50% onscreen gaze data during each of the following events: naming, pretest, test question, test response period. Overall, there were 52/64 valid trials in the trained-word condition (*M* = 3.25, *SD* = 0.86) and 51/64 in the novel-word condition (*M* = 3.19, *SD* = 0.98). For the descriptive data on infants’ attention to the screen, see Table S1 in the Supplemental Materials.

To assess referent selection, we compared how much time infants spent looking at the target (i.e., object targeted by the action) relative to the distractor after hearing the test question. The analysis window corresponded to the 5-second-long test response period and was divided into five 1-second-long time bins. Complementary analyses were carried out on the fine-grained time-course data obtained by grouping the raw data into 50 ms bins.

The beginning of the analysis window was determined following an established procedure (Yin & Csibra, 2015) and coincided with the disappearance of the central attention getter displayed between the objects during the test question. With respect to the onset of the test label, the measurement started on average 437 ms post-onset (*SD* = 60 ms; *Mdn*: 429 ms, Supplemental Materials section SM7. Speech stimuli), which is a little later than typically observed in the infant and toddler word-recognition literature (i.e., 367–400 ms post-onset). Nevertheless, we could capture first saccades made toward objects in response to the test question: on most trials the response period began with infants fixating on the center of the display (Experiment 1: *M* = 65% of valid trials; Experiment 2: *M* = 60%; Experiment 3: *M* = 66%; Experiment 4: *M* = 73%) and it took them on average 404 ms to orient toward an object once the central attention getter disappeared (Supplemental Materials section SM3. First Looks, Figure S1).

The analysis window lasted 5 seconds to provide infants with enough time to respond to the newly introduced words. Processing new words might be slower than familiar word recognition because of differences in their respective cognitive demands (e.g., formation and retrieval of newly formed word-object associations from working memory v. retrieval of familiar words from long-term memory). To date, the few studies looking at 12-month-olds’ online processing of words trained at the lab used different training and testing protocols that are not directly comparable (e.g., Pomiechowska & Gliga, 2019; Tsuji et al., 2020; Yurovsky & Frank, 2017) and none of them involved a central attention getter during labeling. Therefore, we could not derive specific predictions regarding how rapidly infants should respond to words trained in the present task and how quickly the differences between conditions should emerge.

Because infants’ looking responses are sometimes short-lived (for meta-analyses, Bergelson, 2020; Zettersten et al., 2021), following Yin and Csibra (2015), we partitioned the analysis window into five 1-second-long time bins (bin 1: 0–1 s, bin 2: 1–2 s, bin 3: 2–3 s, bin 4: 3–4 s, bin 5: 4–5 s). We derived a separate *looking time difference score* for each time bin: *lookdiffscore* = (*T* − *D*)/(*T* + *D*); *T*: total target looking; *D*: total distractor looking (Supplemental Materials section SM6. Derivation of *lookdiffscore*). *Lookdiffscore* ranges from −1 to 1 and is compared to a chance-level of 0. Positive values indicate longer looking at the target; negative values indicate longer looking at the distractor. A pattern of results in which difference scores recorded in the trained-word condition are positive and significantly higher than those recorded in the novel-word condition would indicate successful referent selection and word mapping.

The mean *lookdiffscores*, computed within participants for each time bin within each condition (trained v. novel word) were used as an outcome variable for linear mixed-effect models. The predictors were condition (a categorical factor with two levels: trained and novel word, referring to what word infants heard at test, within-participant) and time (an ordered category capturing time bin during the test measurement period, within-participant). Intercepts for subjects were used as random effects. For each experiment, we tested for effects of condition and time by comparing a baseline model, *lookdiffscore* ∼ *Condition* + (1|*Subject*), to a model involving an additive relationship between condition and time, *lookdiffscore* ∼ *Condition* + *Time* + (1|Subject), and by comparing the additive model to a model including an interaction between these predictors, *lookdiffscore* ∼ *Condition* * *Time* + (1|*Subject*). The trained-word condition was treated as the reference and parameters were estimated for the novel-word condition. Simple significance tests (i.e., one-sample *t* tests) were used to compare the average difference scores to chance (0).

Additionally, to ensure that the coarse division of the analysis window into five distinct time bins did not obscure more fine-grained differences across conditions, we analyzed infants’ looking patterns using growth curve analysis. The data were aggregated into 50-ms bins, empirical logit transformation was applied to the *proportion of target looking* in each bin (*ptl* = *T*/*(T* + *D*); *T*: total target looking; *D*: total distractor looking; *elog* = *log*[(*ptl* + 0.5)/(*n* − *ptl* + 0.5)]; *n*: total number of samples in a given bin), and the time course of looking was modeled with four orthogonal polynomial time terms (e.g., Garrison et al., 2020; Mahr et al., 2015), examining for their interaction with condition. We included intercepts and slopes for subjects as random effects (after Garrison et al., 2020). The model took the following form: *elog* ∼ *Condition* * (*ot*1 + *ot*2 + *ot*3 + *ot*4) + (1 + *ot*1 + *ot*2 + *ot*3 + *ot*4|*Subject*). The trained-word condition served as the baseline. The normal approximation was used to assess statistical significance for individual parameter estimates.

## RESULTS AND DISCUSSION

**Figure 2.**
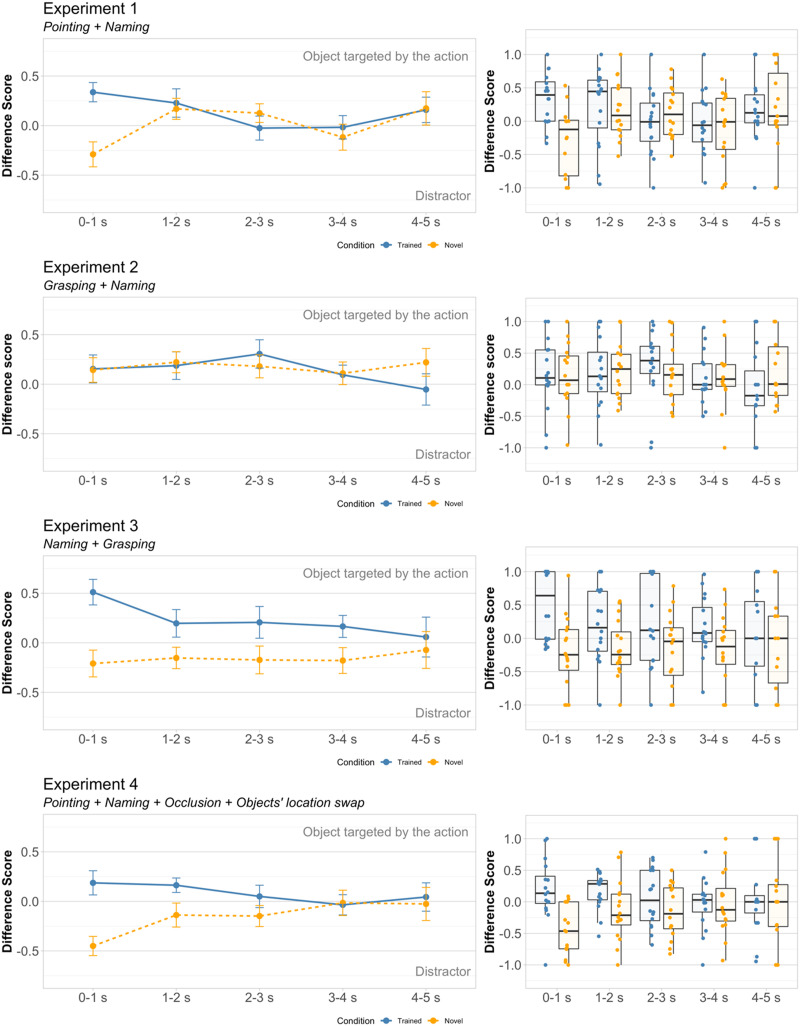
**Difference scores measured in Experiments 1–4 as a function of condition (trained word v. novel word).** For illustration, the data are split by test bin (bin 1: 0–1 s; bin 2: 1–2 s; bin 3: 2–3 s; bin 4: 3–4 s; bin 5: 4–5 s). *Line plots*: Dots indicate means and error bars indicate standard error of the mean. *Box plots*: Black horizontal lines indicate medians. The bottom and the top of the boxes represent the first and the third quartiles. Whiskers extend from the middle quartiles to the smallest and largest values within 1.5 times the interquartile range. Dots represent the individual means of the contributed difference scores across trials within each condition.

The model comparison indicated that most variance in our dataset was explained by including an interaction term between condition and time. The summary of the final model is provided in Table 1. To explore the interaction between condition and time we fit separate *lookdiffscore* ∼ *Condition* + (1|*Subject*) models on the data from each time bin. These analyses revealed that infants responded differently to trained and novel words only during the first bin (corresponding to the first second following the offset of the test question), *β* = −0.628, *t* = −4.349, *p* < .001, 95% CI = [−0.92, −0.33], *d* = 1.05. Upon hearing trained words, they oriented toward the target objects previously singled out by pointing (*M* = 0.34, *SD* = 0.39; Figure 2), while upon hearing novel words, they oriented toward the distractor objects (*M* = −0.29, *SD* = 0.51). These looking patterns were significantly different than expected by chance (higher than chance in the trained-word condition: *t*(15) = 3.489, *p* = .003, *d* = 0.87, 95% CI = [0.13, 0.54]; lower than chance in the novel-word condition: *t*(15) = 2.294, *p* = .036, *d* = 0.57, 95% CI = [−0.56, −0.02]). During the remainder of the test, infants’ responses did not differ across conditions nor from chance (*p*s >.125).

**Table 1.** Model Estimates for the Final Linear Mixed-Effects Models Across Experiments 1–3

	*Dependent variable: Difference score*								
	**Fixed effects**	**Estimate**	***SE***	***T***	***df***	**CI**	***p***		
**Experiment 1**	(Intercept)	0.13	0.06	2.12	34.14	0.01–0.26	0.042	Observations	158
	Condition [Novel]	−0.12	0.07	−1.68	142.12	−0.27–0.02	0.095	Marginal *R*^2^	0.12
	Time [linear]	−0.20	0.12	−1.72	142.30	−0.43–0.03	0.088	Conditional *R*^2^	0.21
	Time [quadratic]	0.22	0.11	1.88	142.19	−0.01–0.44	0.062		
	Time [cubic]	0.10	0.11	0.84	142.00	−0.13–0.32	0.403		
	Time [quartic]	−0.06	0.11	−0.54	141.92	−0.29–0.16	0.593		
	Condition [Novel] * Time [linear]	0.40	0.16	2.46	142.33	0.08–0.72	0.015		
	Condition [Novel] * Time [quadratic]	−0.36	0.16	2.21	142.21	−0.68–−0.04	0.029		
	Condition [Novel] * Time [cubic]	0.23	0.16	1.44	142.01	−0.08–0.55	0.141		
	Condition [Novel] * Time [quartic]	0.11	0.16	0.70	141.92	−0.21–0.43	0.487		
**Experiment 2**	(Intercept)	0.14	0.07	2.11	35.17	0.01–0.27	0.042	Observations	157
	Condition [Novel]	0.04	0.08	0.45	141.10	−0.12–0.19	0.652	Marginal *R*^2^	0.00
								Conditional *R*^2^	0.09
**Experiment 3**	(Intercept)	0.239	0.074	3.24	34.873	0.09–0.39	0.003	Observations	154
	Condition [Novel]	−0.403	0.085	−4.75	138.04	−0.57–−0.23	0.000	Marginal *R*^2^	0.12
								Conditional *R*^2^	0.20
**Experiments 1 vs. 2**	(Intercept)	0.34	0.12	2.83	57.79	0.10–0.58	0.006	Observations	64
	Condition [Novel]	−0.63	0.14	−4.54	32.00	−0.91–−0.35	0.000	Marginal *R*^2^	0.21
	Experiment [Experiment 2]	−0.18	0.17	−1.09	57.79	−0.52–0.15	0.282	Conditional *R*^2^	0.44
	Condition [Novel] * Experiment [Experiment 2]	0.62	0.20	3.15	32.00	0.22–1.01	0.004		

*Note*. Linear mixed-effects models including intercepts for subjects as random effects were used in Experiments 1, 2, and 3 and for comparison across Experiments 1 vs. 2. Linear models were used in Experiment 4 and for comparison across Experiments 1 vs. 3, 2 vs. 3 (see the main text for details).

Together, these results suggest that infants identified the objects targeted by pointing as referents and linked them with the co-occurring words, albeit their looking response was short-lived. Such temporal profile of response conforms with the dynamics of infants’ looking behavior reported in the looking-while-listening tasks (e.g., Schafer & Plunkett, 1998; Tsuji et al., 2020). A supplementary analysis of infants’ first looks after labeling indicated that their word mapping and ensuing referent selection were also evident at the level of first saccades executed in response to the test words (see Supplemental Materials section SM3. First Looks).

Interestingly, upon hearing the novel words, infants oriented toward the nameless distractor objects. This behavior is consistent with employing the mutual-exclusivity principle (i.e., the expectation that distinct names refer to different objects; Markman, 1990) to infer the referents of the novel words by discarding target objects already associated with the trained words (for further evidence of mutual-exclusivity effects at this age, see Pomiechowska et al., 2021; Yin & Csibra, 2015). The ability to link novel untrained words to distractor objects is informative about the nature of representations that infants set up for the trained words that accompanied pointing. Namely, these representations must have been richer (e.g., referential, symbolic, or lexical) than auditory percepts, as these would not license inferences about the denotation of novel words.

**Figure 3.**
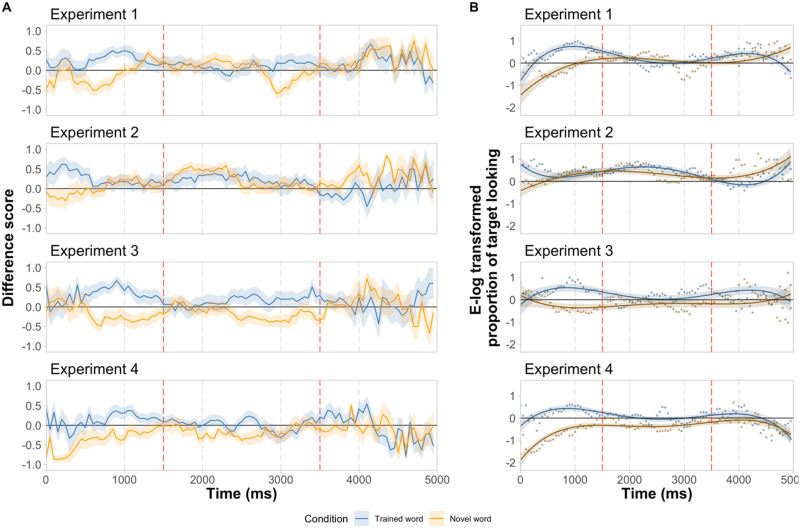
**Time course of looking responses.** (**A**) Evolution of mean looking time differences scores during the test measurement period. (**B**) Results of growth curve analysis. Points represent mean value of empirical logit transformed proportion of target looking over 50 ms; bins and lines represent model estimates; ribbons indicate standard error. (**A–B**) Time 0 is the beginning of the test measurement period (0–5,000 ms), that is, it corresponds to the offset of the test question (e.g., “Where is the mize?”) and offset of the central attention getter displayed between the objects during the test question. The dotted red lines indicate the onset of two additional utterances of the tested label (e.g., “Mize!”, at 1.5 and 3.5 s after the beginning of the test measurement period). Note that at the beginning of the measurement period infants continued to look at the center of the display even though the attention getter was no longer visible (see Data Analysis section and Supplemental Materials section SM3. First Looks, Figure S1), hence fewer data points contributed to the early parts of the looking curves. Chance looking (0) is indicated by a thick black line.

The fine-grained time course of infants’ looking behavior during the test measurement period is depicted in Figure 3. The growth-curve analysis yielded a similar pattern of results as our main analysis: there were main effects of condition, cubic and quartic time terms, as well as interaction effects with the linear, cubic, and quartic time terms (see Table 2 for model summary). More descriptively, infants responded differentially to trained and novel words shortly after labeling. They seemed to switch between the objects more upon hearing the novel words, as suggested by the inflections in the looking curve in this condition.

**Table 2.** Fixed Effects for Growth Curve Models for All Experiments

	*Dependent variable: Difference score*					
	**Fixed effects**	**Estimate**	***SE***	***T***	***df***	***p***
**Experiment 1**	(Intercept)	0.13	0.08	1.67	14.52	0.117
	Condition [Novel]	0.25	0.05	5.03	6091.55	0.000
	Time 1	1.13	0.62	1.81	11.46	0.097
	Time 2	−1.08	0.89	−1.22	11.97	0.245
	Time 3	1.81	0.61	2.95	13.40	0.011
	Time 4	−1.39	0.50	−2.77	9.59	0.021
	Condition [Novel] * Time 1	−3.33	0.59	−5.62	5655.41	0.000
	Condition [Novel] * Time 2	0.93	0.58	1.60	5264.47	0.110
	Condition [Novel] * Time 3	−1.21	0.52	−2.31	5721.69	0.021
	Condition [Novel] * Time 4	−2.54	0.52	−4.88	5639.57	0.000
**Experiment 2**	(Intercept)	0.31	0.09	3.34	14.67	0.005
	Condition [Novel]	0.02	0.05	0.45	4913.48	0.656
	Time 1	0.24	0.78	0.31	13.54	0.759
	Time 2	−0.33	0.69	−0.48	14.91	0.637
	Time 3	1.50	0.59	2.56	15.64	0.021
	Time 4	1.27	0.76	1.68	14.53	0.115
	Condition [Novel] * Time 1	−2.39	0.62	−3.82	3991.62	0.000
	Condition [Novel] * Time 2	−0.27	0.62	−0.44	3470.07	0.661
	Condition [Novel] * Time 3	−1.23	0.55	−2.24	4306.79	0.025
	Condition [Novel] * Time 4	1.91	0.53	3.57	4913.92	0.000
**Experiment 3**	(Intercept)	0.03	0.11	0.30	15.21	0.765
	Condition [Novel]	0.43	0.06	7.30	2768.59	0.000
	Time 1	0.19	0.87	0.21	13.41	0.835
	Time 2	0.58	0.75	0.78	13.61	0.451
	Time 3	0.06	0.96	0.06	16.32	0.951
	Time 4	−0.45	0.88	−0.51	16.12	0.618
	Condition [Novel] * Time 1	−0.83	0.74	−1.12	1767.46	0.263
	Condition [Novel] * Time 2	−0.57	0.73	−0.79	1715.99	0.432
	Condition [Novel] * Time 3	0.72	0.63	1.14	2733.76	0.254
	Condition [Novel] * Time 4	−2.54	0.61	−4.13	4279.75	0.000
**Experiment 4**	(Intercept)	−0.18	0.07	−2.50	15.59	0.024
	Condition [Novel]	0.51	0.05	10.38	5293.12	0.000
	Time 1	0.49	0.52	0.94	14.26	0.361
	Time 2	−1.45	0.43	−3.36	16.03	0.004
	Time 3	0.31	0.65	0.47	13.99	0.645
	Time 4	−1.78	0.69	−2.60	15.12	0.020
	Condition [Novel] * Time 1	−3.54	0.58	−6.06	4076.63	0.000
	Condition [Novel] * Time 2	1.25	0.57	2.21	4173.76	0.027
	Condition [Novel] * Time 3	−0.93	0.51	−1.82	5476.24	0.068
	Condition [Novel] * Time 4	−0.30	0.51	−0.60	6189.40	0.550

*Note*. Random effects for all models are (1 + Time1 + Time2 + Time3 + Time4 | Subject; see Data Analysis section for details).

## EXPERIMENT 2

Experiment 1 provided evidence that infants linked the objects highlighted by pointing with the subsequently provided words. Experiment 2 investigated what process led to this outcome: a communicative-referential interpretation of pointing or a pure association between an object and a word heard when this object was highlighted by an external stimulus. We modified the task from Experiment 1 by replacing pointing actions with grasping actions matched in duration and gross kinematics. If referent selection relies on interpreting the observed object-directed action as communicative, infants should not link the co-occurring label to the object targeted by grasping. This is because, absent further evidence, grasping is interpreted by infants as an instrumental means toward object retrieval (e.g., Woodward, 1998), hence not directly related to the ambient speech. If, on the other hand, infants’ performance is underlaid by associative learning, they should map new words to the objects highlighted by grasping as readily as when the objects were highlighted by pointing.

## METHODS

### Participants

Sixteen infants participated in this experiment (mean age: 12 months 10 days; range: 11 months 15 days to 12 months 26 days). An additional eight infants were tested but excluded from the final analysis due to a failure to complete the task (*n* = 4), crying (*n* = 1), or not providing enough valid data (*n* = 3).

### Stimuli, Design, and Procedure

The stimuli, design, and procedure were the same as in Experiment 1 except for the object-directed action during training: pointing actions were replaced with grasping actions matched in speed and duration. Each grasping action resulted in direct contact between the hand and the target object, maintained throughout the naming event, but was carried out in such a way that the hand did not obscure the object (Figure 1).

### Data Analysis

We followed the same analysis procedure as in Experiment 1. Infants contributed a total of 48/64 valid trials (*M* = 3.00, *SD* = 0.82) in the trained-word condition and 53/64 valid trials (*M* = 3.31, *SD* = 0.95) in the novel-word condition.

## RESULTS AND DISCUSSION

The average difference score data were analyzed using the baseline model: *lookdiffscore* ∼ *Condition* + (1|*Subject*). The model comparison did not justify including time in an additive or an interaction term, *p*s > .55. We found no evidence for significant differences in looking patterns between conditions or time bins (Table 1), a pattern mirrored by infants’ first looks (Supplemental Materials section SM3. First Looks). Therefore, for comparisons to chance (0), we collapsed the data across time. Upon hearing any label, trained or novel alike, infants oriented toward the objects that were previously grasped (Figures 2–3). Their responses were significantly different from chance in the novel-word condition (*M* = 0.15, *SD* = 0.27, *t*(15) = 2.299, *p* = .036, *d* = 0.57, 95% CI = [0.01, 0.29]), but not in the trained-word condition: *M* = 0.15, *SD* = .33, *t*(15) = 1.742, *p* = .102, *d* = 0.41, 95% CI = [−0.03, 0.32]. The growth-curve model confirmed that there was no main effect of condition but revealed significant interactions of condition with linear, cubic and quartic time terms indicating differences in slope and shape of the looking curves across conditions (Table 2). Descriptively, in the trained-word condition infants appeared somewhat faster to orient to the grasped object than in the novel-word condition.

Two aspects of these results should be highlighted. First, the same patterns of responses to trained and novel words demonstrate that infants failed to establish reliable mappings between trained words and grasped objects. Spatiotemporal contiguity between the grasping action and the grasped object was insufficient for infants to associate this object with the novel label that accompanied the action. Upon observing an instrumental action during training infants inspected the targeted object (see Supplemental Materials sections SM4. Attention During Naming; SM5. Attention During Pretest), but seemingly without ascribing it the role of referent that could mediate the formation of a word-object mapping.

Second, orienting toward the grasped object for both types of words suggests that even though no referent ascription or word mapping occurred, infants learned something. Namely, they likely conceived the target object as involved in the goal of the grasping action. This goal representation stored in their working memory guided their looking at test, resulting in their tendency to focus on the objects previously targeted by grasping, irrespective of the words they heard.

### Comparison Between Experiments 1 and 2

To compare infants’ word-mapping performance across experiments we focused on the first second of the test phase, a period during which they displayed evidence of referent selection in Experiment 1. We used a linear-mixed model with two categorical fixed-effects predictors: condition (trained word v. novel word) and experiment (Experiment 1 v. Experiment 2), and intercepts for subjects as random effects: *lookdiffscore* ∼ *Condition* * *Experiment* + (1|*Subject*). Table 1 provides the model summary, indicating a significant main effect of condition and a significant interaction between condition and experiment. Experimental manipulation, being exposed to pointing or grasping during the trial’s training phase, influenced differently looking patterns in response to novel words, with infants in Experiment 2 orienting toward the objects target by the action, *β* = 0.616, *t* = 3.151, *p* = .004, 95% CI = [0.22, 1.01] (see Table 1 for the full model specification). These results confirm that pointing and grasping had different effects on referent selection.

Overall, Experiments 1 and 2 jointly demonstrate that the type of action observed before hearing a novel label critically influenced whether infants established a referential link between this label and the object singled out by the action. To disambiguate novel words in the current task, they prioritized the use of sociopragmatic information resulting from action interpretation over spatiotemporal regularities. More specifically, only communicative interpretation of pointing prompted infants to consider the targeted objects as referents and associate them with the concurrent words. In contrast, grasping, understood by one-year-olds as an instrumental action performed to get access to particular goal objects (e.g., Woodward, 1998), did not facilitate mapping the word that accompanied the grasping action to the grasped object. Instrumental interpretation of object-directed actions licenses inferences about goal states and not reference (Csibra, 2003), hence providing no grounds for linking the grasped objects and the subsequent speech.

Importantly, the diverging results across experiments were not due to differences in attention during training: infants attended equally to the grasped and pointed locations during naming (see Supplemental Materials section SM4. Attention During Naming) or immediately after naming (SM5. Attention During Pretest). Furthermore, it is noteworthy that the pattern of looking time difference scores recorded in response to novel words reversed across experiments. Following pointing, infants displayed negative difference scores indicating a preference for distractor objects, while following grasping they displayed positive difference scores indicating a preference for target objects. This qualitative shift in visual preferences further corroborates the idea that different cognitive mechanisms regulated infants’ responses across experiments, reference ascription in Experiment 1 and goal ascription in Experiment 2.

What did infants make of the speech following instrumental grasping in Experiment 2? While our results suggest that they considered speech irrelevant to the objects that were grasped, at least two other possibilities remain open. First, although instrumental action interpretation does not inform referent selection, it likely does not prevent statistical and associative word-learning mechanisms from working. Therefore, infants in the current task might have nevertheless engaged in bottom-up word learning but did not receive enough exposure during training (e.g., not enough target word repetitions, not enough time observing the objects) to form stable links between the target objects and trained words. Second, they might have considered the new words in relation to the observed action. Given that by 12 months of age infants display sensitivity to morphosyntactic properties of words (Kedar et al., 2017; Waxman & Booth, 2003), it is unlikely that novel labels presented in noun carrier phrases were taken to be verbs. Rather, infants might have attempted to construe them as action labels. Rudimentary knowledge of abstract action-related words learned outside of the lab can be captured between 10 and 13 months (Bergelson & Swingley, 2013) and 14-month-olds learn labels for action roles (e.g., “chaser”; Yin & Csibra, 2015). Future research shall shed light on the question of whether young infants consider instrumental actions as referents of concurrent words.

## EXPERIMENT 3

The findings of Experiments 1 and 2 suggest that 12-month-olds spontaneously interpret pointing, but not grasping, as a communicative act, which in turn leads them to selectively look for referents when new words co-occur with pointing. It is important to note, however, that actions that are typically carried out toward instrumental goals can be repurposed toward communicative goals—as long as other accompanying signals indicate this purpose (e.g., by explicitly addressing one’s audience in action demonstration contexts; Futó et al., 2010; Hernik & Csibra, 2015; Pomiechowska & Csibra, 2017).

In Experiment 3 we tested whether we could trigger a communicative interpretation of grasping and make infants use it in the referent search process. We operationalized this idea by modifying the order of events in our task: we presented the speech stimuli *before* the execution of the grasping action (and not *after*, as in Experiment 2). Infant-directed speech is an ostensive signal itself (Csibra, 2010; Sirri et al., 2020; Zangl & Mills, 2007) and could lead infants to expect subsequent actions to be communicative (Okumura et al., 2013a; Senju & Csibra, 2008). Although the change of order between speech and action seems subtle, we predicted that it should be sufficient to influence the infants’ take on the grasping action, because the initial ostensive signal could facilitate a communicative, and inhibit an instrumental, interpretation of the subsequent action (see also Baker et al., 2009).

## METHODS

### Participants

The final sample consisted of 16 participants (mean: 12 months 3 days; range: 11 months 15 days to 12 months 28 days). Three additional infants were tested but failed to complete the task.

### Stimuli, Design, and Procedure

We used the same stimuli and general procedure as before. The design was modified such that speech preceded grasping during the naming event. First, the objects were shortly presented in silence (.5 s). Then, the naming stimuli, identical to those of Experiments 1 and 2, were played (5 s). Only at the offset of speech, a hand appeared above one of the objects and reached downward to grasp it (2 s). The grasp was presented for 2 seconds, after which the hand released the object and moved upward, leaving the display (1 s). The grasping actions were identical to those of Experiment 2. The subsequent word-mapping test had the same structure as in Experiments 1–2 (Figure 1).

### Data Analysis

To accommodate the modification in the design, we amended our trial inclusion criteria: a trial was considered valid if infants provided a minimum of 50% onscreen data during each of the following events: (a) action, (b) pretest, (c) test question, and (d) test response period. We recorded 45 valid trials (*M* = 2.81, *SD* = 0.91) in the trained-word condition and 47 valid trials (*M* = 2.94, *SD* = 0.77) in the novel-word condition. The remainder of the analysis was the same as in Experiments 1–2.

## RESULTS AND DISCUSSION

Difference scores were analyzed using the following linear mixed-effects model: *lookdiffscore* ∼ *Condition* + 1|*Subject*). Including time did not improve the model fit, *p*s > .50. Condition, but not time or their interaction, predicted infants’ looking responses, indicating that they reacted differently to trained and novel words (Figure 2). Upon hearing novel words, infants looked significantly less to the target objects, *β* = −0.403, *t* = −4.726, *p* < .001, 95% CI = [−0.57, −0.23] (see Table 1 for the full specification of the final model). The growth curve analysis yielded a similar pattern of results, revealing a significant main effect of condition (Table 2). More descriptively, having oriented to the objects labeled with trained and novel words, infants appeared to maintain their gaze on them for most of the measurement period.

Further analyses were carried out on the data collapsed across time bins. Trained words led to a preference for the target objects previously highlighted by grasping (*M* = 0.23, *SD* = 0.41), while novel words led to a preference for the distractors (*M* = −0.17, *SD* = 0.29). Comparisons to chance (0) indicated that changes in looking in both conditions were significantly different from chance: higher than chance for the trained words, *t*(15) = 2.815, *p* = .037, *d* = 0.57, 95% CI = [0.02, 0.45]; and lower than chance for the novel words, *t*(15) = 2.359, *p* = .032, *d* = 0.59, 95% CI = [−0.32, −0.02]. This pattern of results, observed also in infants’ first looks (Supplemental Materials section SM3. First Looks), provides evidence that the infants (1) linked the trained words to the grasped objects and (2) inferred that the distractor objects might be referents of the novel words (see also Pomiechowska et al., 2021).

Here, unlike in Experiment 2, infants successfully adopted the target of a grasping action as the referent of the trained word. This outcome was likely due to the change in the temporal order of events between experiments: that is, that the labeling came before, not after, the action. The ostensive signal in the form of infant-directed speech was the first dynamic stimulus infants were exposed to in each trial, and which they could exploit to make sense of the observed events. Because ostensive signals prompt the interpretation of otherwise instrumental actions as communicative and, hence, referential (Futó et al., 2010; Hernik & Csibra, 2015; Király et al., 2013; Marno & Csibra, 2015; Pomiechowska & Csibra, 2017; Senju & Csibra, 2008), the presence of infant-directed speech before the action likely triggered a communicative interpretation of the action, which was then maintained throughout the event. In other words, having experienced an ostensive signal first, infants construed the following grasping action as a deictic gesture. As such, they could then use it to disambiguate the referent of the preceding speech by assuming co-reference between speech and gesture (Gliga & Csibra, 2009).

Why did the order in which an ostensive signal and a normally noncommunicative action were presented matter? Inferences about others’ goals are continuously revised as their behavior unfolds (e.g., Baker et al., 2009; Baker et al., 2017; see also Pomiechowska & Csibra, 2017). Our experiments rested on the assumption, supported by our findings, that infants would settle on the interpretation of the event using the earliest available evidence. In Experiment 2, where speech followed an instrumental action, infants likely adopted the instrumental reading of the events based on the interpretation of the first observed action. Subsequently, they were either not willing to or did not have enough time to reconsider this interpretation upon receiving further information in the form of speech.

### Comparisons Across Experiments (1 vs. 3, 2 vs. 3)

To investigate whether grasping embedded in a communicative context (Experiment 3) affected infants’ word-mapping performance differently than pointing (Experiment 1) or instrumental grasping (Experiment 2), we compared their referent selection performance across experiments. As before we used the first second of the test phase. We addressed this time window because it captured infants’ word-mapping performance in Experiment 1. Because there was not enough variability across subjects to be included as random intercepts, for each comparison we used mixed-model ANOVAs with condition (trained vs. novel word) as a within-subject factor and experiment as a between-subject factor.

The comparison between Experiments 1 and 3 yielded a significant main effect of condition, *F*(1, 30) = 26.668, *p* < .001, *η*_*p*_^2^ = 0.47, other *p*s > .27, showing that infants’ looking responses to test phrases were comparable across these experiments. Whether the training involved pointing or ostensive grasping, infants differentiated trained and novel words at test. The comparison between Experiments 2 and 3 revealed two significant effects: main effect of condition, *F*(1,30) = 7.728, *p* = .007, *η*_*p*_^2^ = 0.22, and an interaction between experiment and condition, *F(1, 30)* = 7.411, *p* = .009, *η*_*p*_^2^ = 0.21, confirming that ostensive grasping had a different effect on word mapping and subsequent word-recognition than instrumental grasping. Overall, instrumental actions preceded by infant-directed speech may be an as suitable and efficient tool for disambiguating unfamiliar words as pointing gestures.

## EXPERIMENT 4

Together, Experiments 1 to 3 demonstrated that 12-month-olds engage their action interpretation system for reference identification. Note, however, that the tasks used in these experiments did not require them to remember anything else but the location of the labeled objects. In Experiment 4, we investigated the contents of the object representations that infants set up to keep track of referents by using a modified version of the task used in Experiment 1. Here, pointing and labeling were followed by a short occlusion, after which the locations of the objects were swapped. If infants map labels onto object representations that contain visual features of the referent, they should reidentify the referent even if it appears at a different location following occlusion. Hence, they should display a similar pattern of looking as in Experiment 1 (i.e., orienting to the object targeted by pointing following the trained word; orienting to the distractor following the novel word). Alternatively, if infants encode the referents using location-based representations lacking visual features (Kibbe, 2015; Leslie et al., 1998; Samuelson et al., 2011, 2017), the pattern of their visual preferences should flip relative to Experiment 1. Namely, the trained word would elicit longer looking to the distractor (placed now at the location of the object targeted by pointing), while the novel word would elicit longer looking toward the target (placed now at the location of the distractor).

## METHODS

### Participants

The final sample consisted of 16 participants (mean age: 12 months 10 days; range: 11 months 18 days to 12 months 28 days). An additional 10 participants were excluded from the analysis because they failed to complete the calibration (*n* = 1), failed to complete the task (*n* = 4), or did not provide enough data (*n* = 5).

### Stimuli, Design, and Procedure

The stimuli and procedure were generally the same as in the previous experiments. The design was modeled on Experiment 1 and extended to include a short occlusion between the training and test phases (Figure 1). After the hand disappeared from the display following the action, a curtain went down (0.55 s) and covered the stage. The objects remained invisible for 3 seconds before the curtain went up (0.55 s), revealing the objects on the opposite sides of the scene to where they had previously been located. A short jingle sound was played half-way through occlusion to maintain the infants’ attention on the screen. As before, a silent pretest period (2 s) preceded the test phase.

### Data Analysis

We applied the same data analysis pipeline as in the previous experiments. Infants provided a total of 55/64 trials in the trained-word condition (*M* = 3.44, *SD* = 0.73) and 55/64 trials in the novel-word condition (*M* = 3.44, *SD* = 0.73). However, unlike before, there was not enough variability between subjects to be included as random intercepts. Therefore, we used a repeated-measures ANOVA with condition (trained v. novel word) and time (0–1 s, 1–2 s, 2–3 s, 3–4 s, 4–5 s) as within-subject factors, and looking time difference scores as output measure.

## RESULTS AND DISCUSSION

There was only a significant main effect of condition, *F*(1, 15) = 8.293, *p* = .012, *η*_*p*_^2^ = 0.36, other *p*s > .16. A significant effect of condition was also observed in the growth curve analysis, along with significant effects of cubic and quartic time terms as well as interactions of condition with linear and cubic time terms. More descriptively, in the trained-word condition infants seemed to be slower to orient to the target and more prone to switch between objects.

Because the main analysis yielded no significant effects of time, we collapsed the difference scores across time bins. Mirroring the results of Experiment 1, the trained words elicited longer looking toward the objects targeted by pointing (*M* = 0.06, *SD* = 0.15), and the novel words elicited longer looking toward the distractor objects (*M* = −0.18, *SD* = 0.25). Infants’ difference scores were significantly different from chance following novel words, *t*(15) = 2.773, *p* = .014, *d* = 0.70, 95% CI = [−0.31, −0.04], but not following the trained ones, *t*(15) = 1.575, *p* = .136, *d* = 0.39, 95% CI = [−0.02, 0.14]. Similar response patterns were manifest in infants’ first looks (Supplemental Materials section SM3. First Looks).

Despite a short occlusion resulting in the swap of object locations, we observed a similar pattern of results as in Experiment 1. The difference scores were positive in response to the trained words and negative in response to the novel words. This finding indicates that infants reidentified the referents of the trained words after the object locations were swapped during occlusion. Therefore, the object representations that infants set up to track referents must have included featural information.

## GENERAL DISCUSSION

Across four experiments we investigated whether infants use sociopragmatic information in the form of nonverbal actions that accompany language to guide their interpretation of novel words. Our findings make three main theoretical contributions. First, Experiments 1 and 2 jointly show that rapid and spontaneous action interpretation processes triggered by the observation of nonverbal actions have tangible consequences for linguistic reference disambiguation and the ensuing formation of new word-object mappings. Twelve-month-olds who heard a novel word in the presence of two unfamiliar objects linked this word to the object that was targeted by a communicative action affording referent ascription (i.e., pointing, Experiment 1), but not to the object that was targeted by an instrumental action affording goal ascription (i.e., grasping, Experiment 2). This process was evidenced by the pattern of infants’ eye movements: when tested with the word that co-occurred with pointing during training (i.e., trained word), infants looked preferentially at the object that was highlighted by the action; when tested with a different novel word, infants tended to orient toward the other object, which had not been acted upon. Conversely, following grasping, infants did not differentiate between the trained and untrained words, but consistently oriented toward the objects previously grasped. This pattern of findings indicates that reference ascription was conditional on interpreting the nonverbal action that preceded naming as communicative. Infants identified objects highlighted by communicative actions, but not those highlighted by instrumental actions, as potential referents and assumed that these actions co-referred with the concurrent words (Gliga & Csibra, 2009).

Second, Experiment 3 conceptually replicated the findings of Experiment 1 and extended its conclusions. The availability of an ostensive signal in the form of infant-directed speech before the observation of grasping enabled infants to use this action for referent selection. This result suggests that the presence of an ostensive signal prompts infants to interpret subsequent actions as communicative and, consequently, consider the objects targeted by these actions as referents. Although we used a single instrumental action (grasping), we expect that infants would reinterpret other transitive actions (e.g., touching, poking, moving, shaking) as referential after ostensive signals.

Third, Experiment 4 provided another replication of Experiment 1 as well as critical insights into the contents of representations that infants set up to keep track of referents. Even though the objects were occluded after training and emerged from the occlusion in different locations, at test infants recognized the referents of the trained labels in their new locations. This success indicates that (1) object representations onto which infants mapped novel words contained sufficient featural information to enable referent reidentification and that (2) this information was seemingly relevant enough to be maintained in working memory when the referent went out of view (see also Pomiechowska & Gliga, 2021).

In sum, our results provide evidence that young infants use nonverbal aspects of communication to find out what others refer to when using novel words and set up mappings between these words and referent objects. In particular, they can recruit their action interpretation skills, which predate their mastery of language, to decode the nonverbal social information that accompanies verbal communication and use this information to bind lexical entities to object representations. This process is fast, requiring no more than a single-trial exposure to the novel word accompanied by a communicative action. Our findings do not entail that purely associative and statistical learning mechanisms do not contribute to word learning. Rather, it is likely that in contexts where co-occurrences between words and objects are sporadic and short-lived, such as those of the present experiment and also of many naturalistic parent–child interactions (Gleitman & Trueswell, 2020), action interpretation provides preverbal infants with a powerful cognitive shortcut to uncover referential relations between symbols and objects.

Which aspects of action knowledge do infants recruit to make sense of new words? Woodward (2000) suggested that it is the understanding of relational structure of actions that provides infants with word-world mappings. From early on, infants appreciate that object-directed actions express meaningful relations between agents and the targeted objects (e.g., Liu et al., 2019; Skerry et al., 2013; Woodward, 1998, 1999). Hence, action analysis could serve as a filter for potential referents. Analyzing an action as object-directed could make infants flag the target object as a likely referent, while discarding other objects present in the environment from consideration. Our findings, however, speak against this proposal, since pointing and grasping, despite both being object-directed and transitive, had different effects on referent selection. Instead, we believe that it is the inferred purpose of the observed action that prompted (or inhibited) the ascription of referential relations.

We propose that infants have access to (at least) two distinct classes of action interpretation mechanisms, each serving a different function (cf. Csibra, 2003; see also Gergely & Jacob, 2012): one to interpret communicative actions (Csibra, 2010; Sperber & Wilson, 2002) and the other to interpret instrumental actions (Gergely & Csibra, 2003). The operation of each system is triggered by separate sets of cues (e.g., ostensive signals vs. agency cues) and leads to qualitatively different representations of the environment: objects highlighted via communicative actions are taken as potential referents of communicated information, whereas objects highlighted by instrumental actions are construed as part of the goal to be realized. The ensuing representations support inferences about different domains of human activity.

Communicative action interpretation does not necessarily require reasoning about the mental states of the communicator. In infants, it can be triggered by ostensive signals, such as eye contact, infant-directed speech, or contingent reactions (Csibra, 2010). Gradually, with learning and social experience, the set of behaviors that are understood as communicative and, hence, referential, extends to cover a range of typical communicative actions from the human repertoire (talking, pointing, gesturing, drawing, etc.). Our findings indicate that infants interpret pointing as communicative by one year of age even in the absence of preceding ostensive signals. However, other actions that do not normally serve communicative functions, such as grasping, require ostensive signals to be (re)interpreted as referential.

Our findings also speak to the debates on the nature of early words (for a review, Waxman & Gelman, 2009). By one year of age, infants expect words to be more than just sounds that correlate with objects. Rather, they treat words as symbolic referential devices whose function is to single out objects and concepts in communication. Words are part of a system that supports inferences about reference and meaning, such as mutual exclusivity (Markman, 1990). This idea is corroborated by our finding that infants in our experiments went beyond selecting the referents of the words introduced with communicative actions and linking the two together. They used these symbol-referent mappings productively to identify also the referents of completely novel, previously unheard, words. This likely happened by discarding the object onto which they had already mapped a label as a potential referent of the novel label. This finding is in line with recent work demonstrating that infants at this age are equipped with the necessary (lexical, pragmatic, and/or logical) inferential tools to perform mutual-exclusivity computations during referent search (Pomiechowska et al., 2021). That infants made such inferences in our study was probably due to the fact that the communicative action made the local word-referent mapping explicit for them. In more traditional mutual-exclusivity studies, which report that disambiguating novel words using mutual-exclusivity inferences does not emerge before 16–17 months of age (e.g., Halberda, 2003), children are expected to spontaneously recover the lexical item linked to a familiar object.

Finally, we would like to discuss two methodological issues. First, we acknowledge that although the current sample size has been determined based on the previous looking-while-listening studies investigating online word processing in 12- to 14-month-olds and proved sufficient to detect word mapping (Experiment 1, 3, 4) and goal encoding (Experiment 2) in the present work, it might carry limitations. In particular, we would like to acknowledge that small sample sizes might result in overestimating effect sizes (Bergmann et al., 2018; Button et al., 2013) and render replication attempts difficult. Most of the current analyses yield medium-to-large effect sizes. If the true population effect size is large, then a sample size of 16 should be sufficient to replicate it (e.g., providing 80% power using an α of 0.05 in comparison against chance, assuming Cohen’s *d* = 0.80). Note also that graphical analysis of the current data shows that infants’ responses were strongest approximately between 500 and 1,500 ms, while the last second at of the test recording was very noisy, a pattern likely caused by a decline in sustained visual attention over time. Therefore, future studies applying the current paradigm might consider shortening the allocated response time or selecting a shorter analysis time window. The estimates of effect sizes can be selected accordingly (all data are available at https://osf.io/f4q62/).

Second, note that we presented infants with new words and new object pairs on each trial, and their referent selection was tested shortly after. We employed this methodology for two reasons: first, stimulus variability was meant to maximize infants’ interest in the procedure; second, the temporal proximity of training and test was meant to minimize the memory resources required for successful reidentification of the referents and maximize the chance of retention of the ensuing symbol-referent mappings that can be disrupted very easily at this age (Yurovsky & Frank, 2017). Infants were not required to encode the newly formed mappings in their long-term memory, only to maintain them for a couple of seconds in working memory. That is, the current design was tailored specifically to address our interest in the links between action interpretation and referent selection, and our findings do not indicate whether the newly formed word-object mappings were integrated into long-term semantic knowledge systems. Whether such minimal exposure would be sufficient for supporting the retention of word-object mappings beyond the actual trial remains a question for future investigation.

In conclusion, by 12 months of age, infants can use action interpretation to identify referents of novel linguistic expressions and establish links between these expressions and objects. Nonverbal object-directed actions that infants understand as communicative—either because they have already acquired this interpretation (such as pointing) or because the action takes place in an ostensive context (such as communicative grasping)—but not those that are given an instrumental interpretation (such as instrumental grasping) make them conceptualize the targeted objects as referents. This, in turn, promotes the formation of mappings between these objects and novel words taken to co-refer with nonverbal communicative acts. Therefore, infants’ word learning might be assisted by rudimentary sociopragmatic inferences supplied by their action interpretation skills.

## ACKNOWLEDGMENTS

We thank Dora Kampis for help with recording the speech stimuli; Denis Tatone for help with recording the video stimuli and comments about the manuscript; Teodora Gliga, Barbu Revencu, Laura Schlingloff, Maayan Stavans, and anonymous reviewers for comments about the manuscript; Péter Rácz and Oana Stanciu for statistical advice; Levente Madarász for transcribing Hungarian pseudowords in IPA; Krisztina Andrási, Eszter Körtvélyesi, Mária Tóth, and Ágnes Volein for help with participant recruitment and testing.

## FUNDING INFORMATION

GC, H2020 European Research Council (https://dx.doi.org/10.13039/100010663), Award ID: 742231.

## AUTHOR CONTRIBUTIONS

BP: Conceptualization: Equal; Formal analysis: Lead; Methodology: Lead; Visualization: Lead; Writing - Original Draft: Lead; Writing - Review & Editing: Equal. GC: Conceptualization: Equal; Formal analysis: Supporting; Methodology: Supporting; Visualization: Supporting; Writing - Original Draft: Supporting; Writing - Review & Editing: Equal.

## Note

^1^ All participants who completed the task successfully triggered the onset of the test question by fixating on the attention getter. However, not all maintained their attention onscreen during the test questions (please see Table S1 in the Supplemental Materials).

## Supplemental Materials

https://osf.io/f4q62/
