# Supplementary material for: Nonverbal Action Interpretation Guides Novel Word Disambiguation in 12-Month-Olds
Source: Open Mind (Camb). 2022 Jul 1;6:51–76. doi: 10.1162/opmi_a_00055 (PMC9692059; doi:10.1162/opmi_a_00055)
Supplement: Supplementary file 1 [file opmi-06-51-s001.pdf]

Nonverbal action interpretation guides novel word disambiguation in 12-month-olds

Supplementary Materials

Barbara Pomiechowska [1], Gergely Csibra [1,2]

[1] Cognitive Development Center, Department of Cognitive Science, Central European University, [2] Birkbeck College, University of London

SM1. Sample size

SM2. Attention to the Screen

SM3. First Looks

SM4. Attention during Naming

SM5. Attention during Pretest

SM6. Derivation of *lookdiffscore*

SM7. Speech Stimuli

SM1. Sample size

The current sample size was determined a priori based on the literature on early word learning. Looking-while-listening studies employing within-participant designs to investigate word recognition and/or word mapping in infants between 12 and 14 months of age used sample sizes ranging between 15 and 18 participants (Yin & Csibra, 2015; Bergelson & Aslin, 2017[1]; Garrison et al., 2020; Pomiechowska & Gliga, 2019) and yielded large effect sizes (*Cohen's d* = 0.84 to 1.45). Following Yin and Csibra (2015) whose design and outcome measure were the closest to the ones used here, we estimated using G*Power 3.1 (Faul et al., 2007) that testing 16 participants per condition in a within-subject design and using an α of 0.05 would be sufficient to provide 80% statistical power to detect a large effect size (*Cohen's d* = 0.80) in comparison against chance. The sample size of 16 was kept constant for all the experiments in this study.

Bergelson, E., & Aslin, R. (2017). Semantic specificity in one-year-olds' word comprehension. *Language Learning and Development*, *13*(4), 481-501. DOI: https://doi.org/10.1080/15475441.2017.1324308

Faul, F., Erdfelder, E., Lang, A. G., & Buchner, A. (2007). G* Power 3: A flexible statistical power analysis program for the social, behavioral, and biomedical sciences. *Behavior Research Methods*, *39*(2), 175-191. DOI: https://doi.org/10.3758/BF03193146

Garrison, H., Baudet, G., Breitfeld, E., Aberman, A., & Bergelson, E. (2020). Familiarity plays a small role in noun comprehension at 12–18 months. *Infancy*, *25*(4), 458-477. DOI: https://doi.org/10.1111/infa.12333

Pomiechowska, B., & Gliga, T. (2019). Lexical acquisition through category matching: 12-month-old infants associate words to visual categories. *Psychological Science*, *30*(2), 288-299. DOI: https://doi.org/10.1177%2F0956797618817506

Yin, J., & Csibra, G. (2015). Concept-based word learning in human infants. *Psychological Science*, *26*(8), 1316-1324. DOI: https://doi.org/10.1177%2F0956797615588753

[1] Studies by Bergelson and Aslin (2017) and Garrison and colleagues (2020) used overall larger sample sizes covering wider age rangers (12 to 20 months); analyses within different age ranges were conducted on groups of 13 to 17 participants.

SM2. Attention to the Screen

Table S1 shows the proportion of time infants spent looking at the screen, separated by different phases of the experiments. These proportions were calculated in three steps: (1) for each participant and each valid trial, the total number of on-screen data points was divided by the total duration of the respective experimental phase; (2) then the resulting values were averaged within-condition for each participant, and last, (3) these were averaged within-condition across participants. Note that in Experiment 3 *naming* occurred before action; the values in the table were computed for the highlighting period that followed naming and during which the hand remained still holding the object.

		Naming*	Pretest	Test question	Test response
Experiment 1	Trained-word	*M* = 93%, *SD* = 8%<br>*Mdn* = 97%<br>*range*: 76% to 100%	M = 93%, SD = 11%<br>*Mdn* = 96%<br>*range*: 56% to 100%	M = 96%, *SD* = 3%<br>*Mdn* = 98%<br>*range*: 87% to 99%	*M* = 86%, *SD* = *11%*<br>*Mdn* = 90%<br>*range*: 62% to 97%
	Novel-word	*M* = 92%, *SD* = 7%<br>*Mdn* = 93%<br>*range*: 72% to 100%	*M* = 88%, *SD* = 14%<br>*Mdn* = *91%*<br>*range*: 55% to 100%	*M* = 95%, *SD* = 8%<br>*Mdn* = *97%*<br>*range*: 68% to 100%	*M* = 91%, *SD* = 8%<br>*Mdn* = 93%<br>*range*: 74% to 100%
Experiment 2	Trained-word	*M* = 92%, *SD* = 11%<br>*Mdn* = 96%<br>*range*: 58% to 100%	*M* = 90%, *SD* = 12%<br>*Mdn* = 96%<br>*range*: 60% to 100%	*M* = 91%, *SD* = 10%<br>*Mdn* = 96%<br>*range*: 66% to 98%	*M* = 88%, *SD* = 10%<br>*Mdn* = 90%<br>*range*: 51% to 99%
	Novel-word	*M* = 89%, *SD* = 15%<br>*Mdn* = 96%<br>*range*: 51% to 100%	*M* = 90%, *SD* = 13%<br>*Mdn* = 94%<br>*range*: 53% to 100%	*M* = 92%, *SD* = 12%<br>*Mdn* = 96%<br>*range*: 55% to 99%	*M* = 86%, *SD* = 13%<br>*Mdn* = 90%<br>*range*: 51% to 99%
Experiment 3	Trained-word	*M* = 91%, *SD* = 11%<br>*Mdn* = 95%<br>*range*: 66% to 100%	*M* = 90%, *SD* = 10%<br>*Mdn* = 94%<br>*range*: 71% to 100%	*M* = 95%, *SD* = 5%<br>*Mdn* = 97%<br>*range*: 80% to 98%	*M* = 83%, *SD* = 12%<br>*Mdn* = 82%<br>*range*: 63% to 99%
	Novel-word	*M* = 90%, *SD* = 11%<br>*Mdn* = 91%<br>*range*: 62% to 100%	*M* = 90%, *SD* = 13%<br>*Mdn* = 97%<br>*range*: 55% to 100%	*M* = 95%, *SD* = 5%<br>*Mdn* = 97%<br>*range*: 80% to 98%	*M* = 82%, *SD* = 10%<br>*Mdn* = 82%<br>*range*: 65% to 97%
Experiment 4	Trained-word	*M* = 94%, *SD* = 6%<br>*Mdn* = 95%<br>*range*: 80 to 99%	*M* = 92%, *SD* = 8%<br>*Mdn* = 94%<br>*range*: 74% to 100%	*M* = 97%, *SD* = 1%<br>*Mdn* = 98%<br>*range*: 95% to 99%	*M* = 89%, *SD* = 8%<br>*Mdn* = 89%<br>*range*: 71% to 98%
	Novel-word	*M* = 95%, *SD* = 3%<br>*Mdn* = 96%<br>*range*: 90 to 100%	*M* = 90%, *SD* = 11%<br>*Mdn* = 94%<br>*range*: 68% to 100%	*M* = 92%, *SD* = 8%<br>*Mdn* = 95%<br>*range*: 72% to 99%	*M* = 89%, *SD* = 7%<br>*Mdn* = 91%<br>*range*: 72% to 99%

Table S1. The amount of attention to the display during valid trials as a function of the phase of trials (naming, pretest, test question, and test response) and condition (trained v. novel-word).

SM3. First Looks

During the test question, infants' gaze was attracted to the center of the display by a dynamic attention getter, the disappearance of which coincided with the offset of the question (i.e., the offset of the label placed at the end of the phrase). To investigate whether the first saccades away from the attention getter towards one of the two objects stimuli reflected referent selection, we calculated the proportion of the saccades directed at the target object following the disappearance of the attention getter. In this calculation, we only used the trials in which during the period extending from the offset of the attention getter displayed during the test question and the first saccade (1) no data were missing (i.e., the gaps between registered data points were not larger than 2 missing samples); and (2) the data were spatially accurate (i.e., AOI hits were not scattered across more than one AOI, as assessed by visual inspection of the trial-by-trial plots; fully automatized analysis not implementing this criterion yielded similar results). This resulted in the exclusion of additional trials relative to the main analysis (Experiment 1: n = 1 due to missing data, n = 3 due to dispersion; Experiment 2: n = 7 due to missing data; n = 4 due to dispersion; Experiment 3: n = 11 due to missing data; n = 2 due to dispersion; Experiment 4: n = 14 due to missing data; n = 3 due to dispersion). Overall, the first looks were available in 357 trials (Experiment 1: n = 49, M = 3.06, SD = 0.93 in the trained-word condition, n = 48, M = 30, SD = 0.80 in the novel-word condition; Experiment 2: n = *43*, M = 2.69, SD = 0.87 in the trained-word condition, n = 47, M = 2.94, SD = 1 in the novel-word condition; Experiment 3: n = 38, M = 2.38, SD = 1.02 in the trained-word condition, n = 39, M = 2.44, SD = 1.03 in the novel-word condition; Experiment 4: n = 46, M = 2.88, SD = 1.02 in the trained-word condition, n = 47, M = 2.94, SD = 0.93 in the novel-word condition).

The patterns of eye movements triggered by the test question mirrored the results of the analysis based on looking duration (Figure S1A). In Experiments 1, 3, and 4, which involved communicative actions, the first looks were distributed differently in response to trained versus

novel words (Experiment 1: Z = -2.321, p = 0.020; Experiment 3: Z = -2.255, p = 0.024; Experiment 4: Z = -2.417, p = 0.016, by Wilcoxon signed-rank tests). Hearing the trained words led infants to saccade towards the target objects (one-sample Wilcoxon signed-rank tests were used to carry out comparisons to chance (.05); Experiment 1: M = 0.69, SD = 0.26, Z = -2.266, p = 0.024; Experiment 3: M = 0.71, SD = 0.31, Z = -2.191, p = 0.029; Experiment 4: M = 0.57; SD = 0.32, Z= -0.734, p = 0.463), while upon hearing the novel words they tended to saccade towards to the distractor objects (Experiment 1: M = 0.43, SD = .28, p = 0.353; Experiment 3: M = 0.42, SD = 0.3, p = 0.436; Experiment 4: M = 0.23; SD = 0.29, Z = -2.615, p = 0.008).

In contrast, in Experiment 2, the only task that involved an instrumental action, the first looks in the trained-word condition did not differ from those in the novel-word condition, t(15) = 0.332, p = 0.745, and remained around chance in both conditions (trained word: M = 0.54, SD = 0.31, p = 0.644; novel word: M = 0.50; SD = 0.33, p = 1).

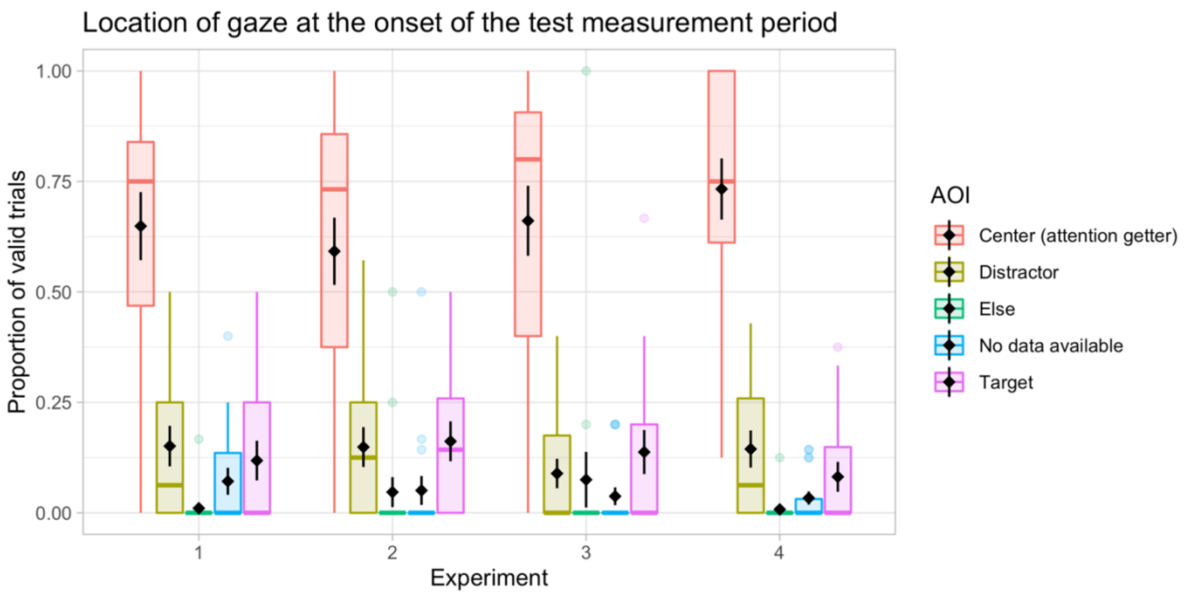

Figure S1. Distribution of attention at the onset of the test measurement period. To assess where infants looked after the offset of the test question, we computed where their gaze was located during the first sample of the test measurement period. For each infant, we derived the mean *proportion of valid trials* for each area of interest (AOI) by dividing the number of trials in which the gaze was recorded in a given AOI by the total number of valid trials. We then averaged these values within experiments. Overall, on most trials infants looked at the center of the display when the test measurement period begun. It took them on average 404 ms to saccade towards an object (Experiment 1: M = 351 ms; Experiment 2: M = 397 ms; Experiment 3: M = 432 ms; Experiment 4: M = 435 ms).

SM4. Attention during Naming

During naming (5 s, or 300 samples) in Experiments 1, 2, and 4, a still hand highlighted the target object either via pointing at it or grasping it. We verified that infants attended equally long to the two objects during this phase by counting the total of target and distractor AOI hits (Experiment 1: M = 189, SD = 48; Experiment 2: M = 220, SD = 71; Experiment 4: M = 178, SD = 61). Then, we assessed infants' attention to the target objects during this period by calculating difference scores between the two AOIs the same way as in the main analysis (Figure S1B). Overall, infants prioritized the objects highlighted by the actions in all three experiments (Experiment 1: M = 0.24, SD = 0.33; Experiment 2: M = 0.42, SD = 0.32; Experiment 4: M = 0.43, SD = 0.31) and their attendance to target AOIs was higher than expected by chance (Experiment 1: $t(15) = 2.869$, $p = .012$, $d = 0.72$, 95% CI = [0.06, 0.42]; Experiment 2: $t(15) = 5.315$, $p < .001$, $d = 1.33$, 95% CI = [0.25, 0.59]; Experiment 4: $t(15) = 5.507$, $p < .001$, $d = 1.377$, 95% CI = [0.26, 0.59]. The patterns of infants looking behavior were comparable across experiments, as revealed by a one-way ANOVA with experiment (1 v. 2 v. 4) as a between-subject factor, $F(2, 45) = 1.803$, $p = 0.177$, $\eta_p^2 = 0.07$. This indicates that the differential word-mapping performance observed in response to pointing (Experiments 1 and 4) and grasping (Experiment 2) was not a result of unequal levels of attention to the locations highlighted by these actions.

We acknowledge that while the pointing hand never touched the object and remained outside of the target AOI, the grasping hand made contact with the object, getting inside the AOI. Therefore, in the case of grasping, we cannot disentangle how long infants looked at the object itself relative to the hand holding it. This, however, does not affect our conclusion that infants prioritized the location highlighted by grasping over the distractor object.

Unlike in Experiments 1, 2, and 4, described above, in Experiment 3, the naming and action were dissociated in time. First, infants heard the naming phrase and then saw one of the

objects being grasped. The hand remained still, maintaining contact with the object for 2 seconds. During this time, infants looked to the target location about twice as much as the distractor (*M* = 0.65, *SD* = 0.41, *t*(15) = 7.497, *p* < .001, *Cohen's d* = 1.87, 95% CI = [0.46, 0.83]).

SM5. Attention during Pretest

To explore how the observed actions affected infants' attention to the objects after the hand disappeared from the display, we quantified their looking behavior during the pretest period (i.e., after the hand moved out from the display and before the dynamic attention getter appeared, marking the beginning of the test phase). As above, we modeled this analysis on our main analysis. We computed looking time difference scores on AOI hits summed over the duration of the pretest (2 s) and entered them into a one-way ANOVA with Experiment (1 to 4) as a between-subject factor. This analysis yielded a significant main effect of experiment, $F(3, 60) = 3.073$, $p < .034$, $\eta_p^2 = 0.13$. Post-hoc comparisons revealed that infants' looking behavior at pretest was significantly different only between Experiments 3 and 4 ($p = 0.035$, other *p*s > 0.09, Tukey corrected). The difference scores were positive in Experiments 1, 2, and 3 (Experiment 1: $M = 0.14$, $SD = 0.40$; Experiment 2: $M = 0.18$, $SD = 0.34$; Experiment 3: $M = 0.23$, $SD = 0.31$) and negative in Experiment 4 ($M = -0.08$, $SD = 0.19$), which suggests that infants had a tendency to look towards the object previously targeted by the action. These results suggest that during the pretest periods, infants tend to focus on the objects previously targeted by the actions and provide further evidence that the differences in word-mapping performance following pointing and instrumental grasping were not due to lower attendance to objects and locations highlighted by grasping.

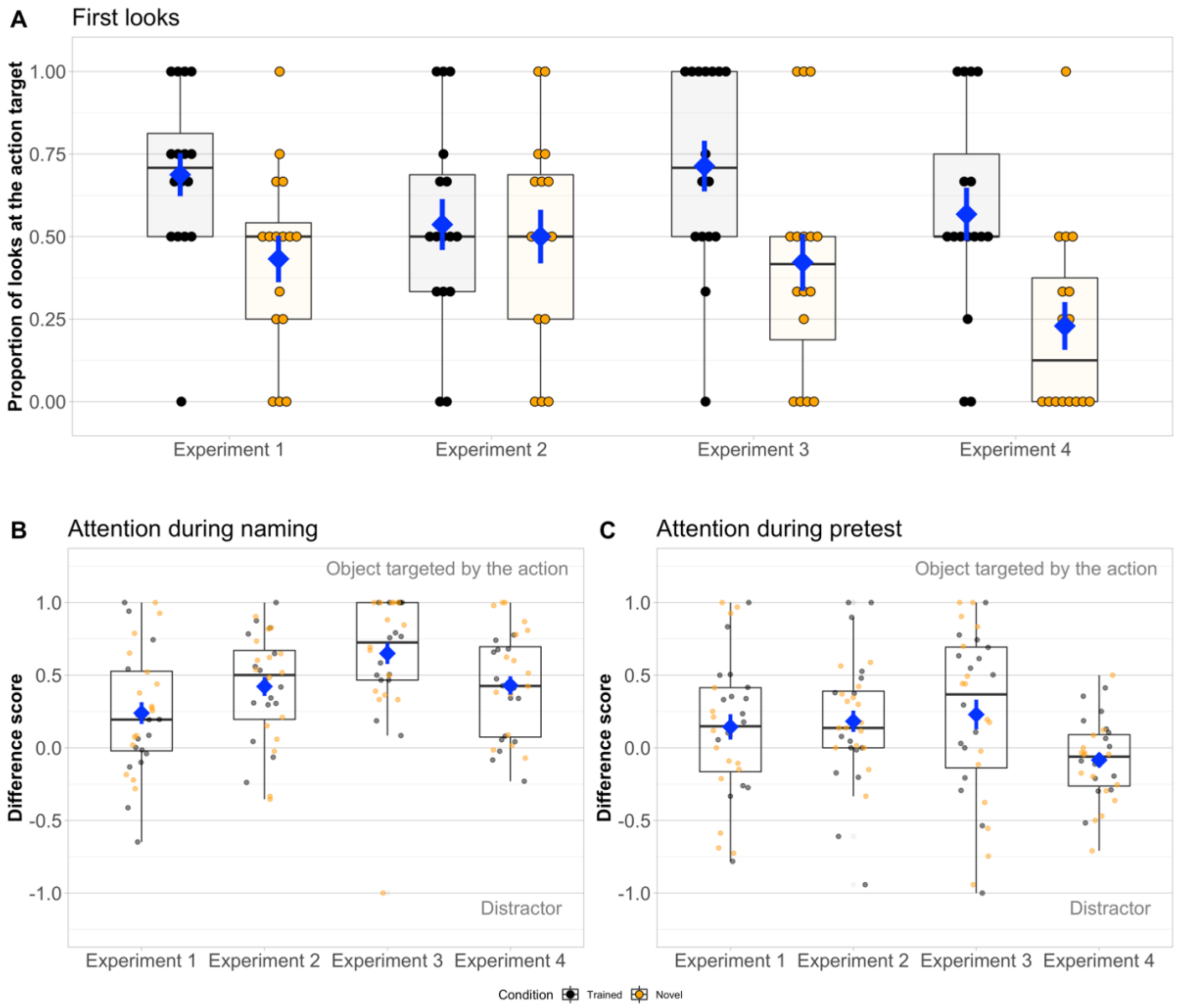

Figure S2. Results of complementary analyses. **A** Proportion of first looks to the object targeted by the action (1 indicates that all first looks were directed at the object targeted by the action; 0 indicates that all first looks were directed at the distractor object). **B** Difference scores during naming (Experiments 1, 2, 4) and during the period when the still hand highlighted the object following naming (Experiment 3). **C** Difference scores during pretest. **A, B, C** Blue diamonds indicate means, and blue bars indicate standard error of the mean. Black horizontal lines indicate medians. The bottom and the top of the boxes represent the first and the third quartiles. Whiskers extend from the middle quartiles to the smallest and largest values within 1.5 times the interquartile range. Dots represent the individual means across trials within each condition.

SM6. Derivation of *lookdiffscore*

To derive looking time difference scores (*lookdiffscore*) from the raw gaze data, we applied the following steps: first, we defined two rectangular areas of interest (AOIs: 805 × 535 px), one encompassing the target object and the other one encompassing the distractor object. Second, we derived the total looking times at the target and distractor for each time bin by summing separately target and distractor AOI hits (i.e., gaze points falling within a given AOI) for each AOI throughout the test response period. Lastly, the total looking time data were used to compute difference scores for each time bin of interest (bin 1: 0-1 s corresponding to 1-60 samples, bin 2: 1-2 s corresponding to: 61-120 samples, bin 3: 2-3 s corresponding to 121-180 samples, bin 4: 3-4 s corresponding to 181-240 samples, bin 5: 4-5 s corresponding to 241-300 samples).

SM7. Speech Stimuli

The labeling phrases presented at test (“*Look! Where is the [LABEL]?*”) were recorded one by one with the constraint to last 2 seconds. The duration of the tested pseudowords varied slightly across phrases. Table S2 summarizes the duration data.

Table S2. Duration of the pseudowords presented in the labeling phrases at test.

**Pseudoword**	**Duration** (ms)
baku	396
bite	370
bóta	481
cefo	518
düpi	425
fego	563
géte	500
gupa	405
kabó	459
kitő	470
mize	461
nala	392
pádu	433
püke	412
tegi	363
toda	347
mean	**437**
median	**429**
std	**60**
